# Mikhail ‘Misha’ Blagosklonny’s enduring legacy in geroscience: the hyperfunction theory and the therapeutic potential of rapamycin

**DOI:** 10.18632/aging.206189

**Published:** 2025-01-12

**Authors:** David A. Barzilai

**Affiliations:** 1 Geneva College of Longevity Science, Geneva 1204, Switzerland; 2 Healthspan Coaching LLC, Barzilai Longevity Consulting, Boston, MA 02111, USA

**Keywords:** rapamycin, longevity medicine, healthspan, geroscience, hyperfunction

## Abstract

The untimely passing of Dr. Mikhail “Misha” Blagosklonny has left a lasting void in geroscience and oncology. This review examines his profound contributions, focusing on his pioneering the Hyperfunction Theory and his advocacy for rapamycin, an mTOR inhibitor, as a therapeutic agent for lifespan extension. Contrary to traditional damage-centric models, the Hyperfunction Theory rejects damage accumulation as the primary driver of aging. Instead, it redefines aging as a quasi-programmed process driven by the persistent, excessive activity of growth-promoting pathways beyond their developmental roles, leading to age-related pathologies. We explore how Blagosklonny’s insights predict rapamycin’s ability to decelerate aging by modulating excessive mTOR signaling, supported by empirical evidence across multiple physiological systems, including immune, cardiovascular, cognitive, and oncologic health. His forward-thinking approach, advocating for the cautious clinical use of rapamycin and suggesting personalized, preventive, and combination therapy strategies, has catalyzed interest in translational geroscience. This review synthesizes Blagosklonny’s legacy, presenting rapamycin as a foundational pharmacological intervention with potential in managing age-related decline and extending healthspan, and underlines his impact in shifting aging research from theoretical frameworks to actionable interventions. Blagosklonny’s work remains an enduring inspiration, paving the way toward treating aging as a modifiable condition.

## INTRODUCTION

The untimely passing of Dr. Mikhail ‘Misha’ Blagosklonny marked the loss of a pioneering scientist—and a valued colleague and friend—who reshaped oncology and geroscience [1–4]. Throughout his career, Blagosklonny authored over 270 publications, served as an editor for Cell Cycle, Oncotarget and Aging (Albany NY), and advanced a more integrated view of cancer, cellular biology, and aging [1]. By bridging these fields, he reframed aging as a quasi-programmed process in which growth-promoting pathways persist beyond their developmental purpose, thereby contributing to age-related diseases. His legacy rests on two major contributions: the Hyperfunction Theory of aging and his pioneering advocacy for rapamycin, an mTOR (mechanistic target of rapamycin) inhibitor, as a therapeutic intervention for extending lifespan. The Hyperfunction Theory represents a fundamental departure from traditional damage-based theories of aging. By framing aging as a quasi-programmatic process driven by the overactivity of growth-promoting pathways such as mTOR, Blagosklonny positioned his work as an alternative to damage accumulation models, which he critiqued as inadequate for explaining the underlying biology of aging.

Blagosklonny’s 2006 proposal that rapamycin might serve as a “longevity drug” anticipated the 2009 Interventions Testing Program (ITP) findings, which confirmed rapamycin’s capacity to extend lifespan in genetically diverse mice, even when administered late in life. This manuscript reviews Blagosklonny’s Hyperfunction Theory, focusing on its implications for rapamycin’s mechanism of action, potential in longevity, and his proposed framework for clinical application.

## Redefining aging: the Hyperfunction Theory

### Hyperfunction versus damage models

Before Blagosklonny’s contributions, aging research was primarily guided by damage-centric theories, which propose that aging is driven by cumulative damage from stressors such as oxidative stress, protein aggregation, DNA degradation, and other factors [5–19]. These theories positioned aging as a process of gradual deterioration over time.

Blagosklonny’s Hyperfunction Theory introduced a complementary perspective, proposing that aging is not only due to accumulated damage but also to the persistent activity of growth-promoting pathways, such as mTOR, beyond their developmental roles [20–22]. The Hyperfunction Theory posits that these growth pathways, which drive development and reproduction in early life, become deleterious when they remain active in later life, leading to cellular hypertrophy, hyperfunction, and senescence [21, 23–27]. Unlike damage-based theories, which attribute aging to the gradual accumulation of molecular and cellular damage, the Hyperfunction Theory explicitly rejects this perspective as the primary explanation for aging [28, 29]. Instead, it proposes that persistent growth-promoting signals, such as mTOR, drive cellular and tissue dysfunction. Blagosklonny likened this to a “runaway car without brakes,” where damage occurs as a secondary consequence of unchecked growth signaling rather than as the root cause [25, 30].

Blagosklonny’s Hyperfunction Theory aligns with the concept of antagonistic pleiotropy, originally proposed by George C. Williams in 1957, which posits that genes beneficial in youth can contribute to aging later in life [31, 32]. Hyperfunctional pathways such as mTOR reflect this concept by supporting survival and reproductive success early on, while driving pathology as organisms age. By expanding on this idea, Blagosklonny integrated damage models with hyperfunction, suggesting that prolonged mTOR signaling exacerbates both cellular overactivity and the accumulation of molecular damage [22, 33]. This alignment underscores the evolutionary roots of aging, where pathways beneficial for growth and reproduction become maladaptive in later life. For instance, David Gems, a key contributor to theoretical and experimental studies of aging, has highlighted the role of mTOR signaling in promoting hypertrophy and fibrosis [34], which contribute to diseases such as atherosclerosis and cancer, and in aging *C. elegans* hermaphrodites, run-on physiological apoptosis becomes a pathogenic with time [35]. His perspective aligns with the Hyperfunction Theory, suggesting that these mechanisms represent a significant aspect of aging biology [20].

## The Hyperfunction Theory vs. damage models: exchange with aubrey de grey

While de Grey emphasizes that cellular damage is the principal driver of aging, Blagosklonny suggests that hyperfunction may underlie much of this damage, establishing a cycle where excessive growth signaling promotes metabolic byproducts that accumulate and cause cellular decline [6, 30].

Blagosklonny directly engaged with Aubrey de Grey, a proponent of damage-based theories, in a 2021 exchange published in Rejuvenation Research. Blagosklonny emphasized that hyperfunction, not damage accumulation, underpins aging, arguing that Hyperfunction Theory explains why damage accumulates—not from aging but as a downstream byproduct of hyperactive signaling:

“Hyperfunction of signaling pathways can occur without progressive changes of their activity. For example, when the same activity of growth-promoting pathways remains unchanged in postdevelopment, it is a hyperfunction. By analogy, a car driving 65 mph on highway is not speeding (hyperfunction) but driving 65 mph on the driveway is. In the latter case, the car certainly will be damaged, but not by rusting (molecular damage), but by damage of its macroparts. Similarly, hyperfunction does not cause molecular damage, but causes organ damage. Thus, the brain is damaged by stroke, which can be a result of hypertension, which, in turn, is developed by hyperfunctional cells of multiple tissues. There is no place for molecular damage in this sequence of events…”

In his rebuttal, de Grey argued that while the Hyperfunction Theory offers valuable insights, damage repair remains essential for addressing aging:

“While hyperfunction undoubtedly contributes to aging, it cannot fully explain the accumulation of oxidative and genetic damage that impairs cellular function [30].*”*

Blagosklonny further posited that while molecular damage accumulates, it does not necessarily constrain lifespan under typical conditions; however, if interventions extend lifespan significantly, such damage may become more limiting [36]. This dialogue highlights the contrasting paradigms while reinforcing Blagosklonny’s central assertion that aging interventions should prioritize targeting hyperfunction at its source.

Building on the Hyperfunction Theory, Blagosklonny proposed that targeting overactive growth pathways could mitigate aging and its associated diseases. This theoretical framework directly informs the exploration of rapamycin, an mTOR inhibitor, as a potential therapeutic agent. The Hyperfunction Theory, together with João Pedro de Magalhães’ related developmental model [37, 38] has inspired the emergence of an expanding suite of programmatic theories, encompassing hypofunction, costly programs, constraint theory, and adaptive death [39–44].

## Predictive health benefits of rapamycin based on the Hyperfunction Theory

Blagosklonny’s insights on cellular aging center around the concept that the transition from a quiescent (non-dividing) state to a senescent one—termed geroconversion—is driven by growth-promoting mediators, notably the mTOR (mechanistic target of rapamycin) pathway, particularly when cells encounter a block in the cell cycle [45]. Under normal circumstances, cells in quiescence remain inactive without progressing to senescence. However, when growth signals such as those from the mTOR pathway remain active in cells that can no longer divide, it results in an overactive cellular state that fosters aging and senescence via continued, maladaptive activity of growth-related pathways beyond their developmental roles [22]. This theory suggests that key molecular mechanisms, including the mTOR pathway, remain chronically overactive, thereby promoting cellular processes that, while beneficial in early life, contribute to age-related diseases as they persist.

Rapamycin, an mTOR inhibitor, offers a promising therapeutic intervention by selectively modulating this excessive signaling, potentially decelerating the aging process. Blagosklonny proposed that if hyperfunction drives aging, then inhibiting these pathways with rapamycin should delay or mitigate multiple age-related conditions [22]. His predictions, derived from the Hyperfunction Theory, have been corroborated by numerous studies exploring rapamycin’s effects across several biological systems [22, 24, 25, 46, 47]. The following sections explore the health benefits of rapamycin as predicted by Blagosklonny and supported by empirical research.

- Immune Function: According to the Hyperfunction Theory, mTOR hyperactivity contributes to immune dysfunction by promoting chronic inflammation. Blagosklonny hypothesized that mTOR inhibition would alleviate immune hyperfunction and rejuvenate immune responses. This prediction was confirmed in animal models [48–52], and subsequently by Joan Mannick and colleagues, who found that elderly patients treated with low-dose everolimus (a rapamycin analog) demonstrated improved vaccine efficacy and reduced infection rates [53–55]. Foundational work by Chen et al. (2009) demonstrated that rapamycin can restore hematopoietic stem cell (HSC) function in aged mice, enhancing adaptive immunity and effective responses to viral challenges [56]. More recently, Ando et al. (2023) showed that mTOR signaling plays a crucial role in regulating T cell exhaustion and the efficacy of PD-1-targeted immunotherapy, revealing the nuanced outcomes of mTOR inhibition depending on the phase of immune activation [57]. While not all findings have been consistent, initial evidence shows promising potential, marking this approach as an area of significant scientific interest [55, 58]. These results support Blagosklonny’s hypothesis that rapamycin can enhance immune function by moderating age-associated immune hyperactivity without compromising essential immune defenses.
- Cardiovascular Health: Persistent mTOR activation is thought to contribute to hypertrophy and fibrosis in cardiovascular tissues, accelerating age-related arterial plaque buildup [59–61]. Blagosklonny proposed that mTOR inhibition could have protective cardiovascular effects [22, 62]. Research on companion dogs, including studies led by Matt Kaeberlein, has yielded promising evidence that rapamycin may reduce markers of cardiac aging and improve heart function [63]. These preliminary findings align with Blagosklonny’s theoretical predictions, though some variability in results suggests that further investigation is necessary to validate and deepen our understanding of these effects [63, 64]. These findings underscore rapamycin’s potential to support cardiovascular health, reducing the impact of age-related pathology and positioning mTOR inhibition as a therapeutic avenue in cardiology [60, 61, 65].
- Cognitive Function: Blagosklonny suggested that by reducing hyperfunction in neural cells, rapamycin could prevent neuroinflammation associated with neurodegenerative diseases [47, 62, 66]. He proposed that rapamycin might help mitigate the buildup of amyloid and tau proteins, hallmarks of Alzheimer’s disease. Studies in rodent models of neurodegeneration have supported this prediction, showing that rapamycin delays cognitive decline, reduces neuroinflammation, and slows the accumulation of amyloid plaques [67–75]. Similarly, in Parkinson’s disease (PD) models, rapamycin mitigates neurodegeneration by inhibiting mTORC1 activity, rescuing dopaminergic neuron loss and behavioral deficits [76]. King et al. (2008) demonstrated that rapamycin also inhibits the aggregation of misfolded proteins, such as polyglutamine and huntingtin, via a reduction in protein synthesis, independent of its effects on autophagy [77]. These findings validate the notion that mTOR inhibition protects cognitive function by reducing cellular hyperfunction and preserving neural health.
- Cancer Prevention: A cornerstone of Blagosklonny’s Hyperfunction Theory is the overlap between aging and cancer, both driven by hyperactive growth pathways [22, 23, 46, 78–84]. Blagosklonny hypothesized that mTOR inhibition would reduce cancer risk by suppressing the excessive cellular signaling that fuels tumor development. Research has confirmed that rapamycin reduces tumor progression and pre-cancerous lesion growth, affirming his view that hyperfunction contributes to both aging and cancer [12, 78, 79, 85–92]. This reinforces rapamycin’s potential as a dual-action therapeutic, addressing cellular pathways that drive both age-related decline and carcinogenesis.

Rapamycin has shown considerable potential for lifespan extension across diverse animal models, from invertebrates such as *C. elegans* [93] and *D. melanogaster* [93] to mammals, including mice, where it mitigates various aging-related phenotypes [80, 85, 87, 88, 94–110]. In mammalian models, rapamycin has been associated with improvements in body composition, metabolic and physical function, and reduced incidence of aging-related pathologies such as sarcopenia, osteoarthritis, ovarian decline, and tendon stiffness. These effects extend across multiple physiological systems—circulatory, respiratory, digestive, musculoskeletal, endocrine, integumentary, reproductive, and oral health—as well as in the management of benign neoplasms and other age-associated conditions [55, 64, 67, 97, 102, 103, 106, 111–125]. Preliminary findings, presented by Adam Salmon at the American Aging Association 52nd Annual Conference (2024), further suggest rapamycin’s potential for lifespan extension in primates. Studies consistently report that rapamycin administration increases median and maximum lifespan [89, 95, 99, 101, 102, 123, 126–128]. These findings have sparked considerable interest in rapamycin’s translatability to human aging, with preliminary data from human studies and clinical trials suggesting beneficial effects on age-related biomarkers and immune function. Such results underscore rapamycin’s emerging role as a pharmacological intervention with broad potential in mitigating age-associated decline in humans. As research continues, there is increasing optimism that rapamycin may hold practical applications for human life extension and age-related disease prevention, validating Blagosklonny’s predictions within the framework of the Hyperfunction Theory.

## Clinical translation and future directions

### Pragmatic use: a balanced approach to rapamycin’s clinical application

Blagosklonny was a strong advocate for rapamycin’s cautious use in clinical settings, arguing that extensive animal safety data and emerging human studies supported its potential benefits in promoting healthy aging. In his 2019 article, “Rapamycin for Longevity,” he proposed that delaying treatment until human lifespan studies are complete would defer possible benefits for individuals who could benefit today [126]. Blagosklonny’s view remains controversial, as many researchers, including those working in the field of geroscience, emphasize the importance of validating efficacy and safety in humans before recommending rapamycin for longevity purposes [129]. Nevertheless, Blagosklonny’s advocacy has catalyzed significant interest and momentum in aging research, sparking increased funding and studies into rapamycin’s applications [32, 58, 64, 71, 72, 95, 110, 120, 130–144]. By championing a “pragmatic use” approach, Blagosklonny has opened pathways for informed, personalized decision-making between patients and healthcare providers. This framework allows patients to weigh the potential benefits and risks of off-label rapamycin use, guided by ongoing research and medical supervision, while awaiting definitive evidence on lifespan extension effects in humans.

While Blagosklonny advocated for rapamycin’s use based on its established safety profile when administered appropriately, he acknowledged discussions in the scientific community about potential side effects and the exploration of alternatives. Some researchers have proposed that developing TORC1-specific inhibitors or utilizing alternative rapalogs like everolimus could mitigate rapamycin’s side effects, such as glucose intolerance and immunosuppression [119]. Studies by Lamming et al. (2013) suggest that these rapalogs may offer similar therapeutic benefits with reduced adverse effects due to their different pharmacokinetic properties [119]. Additionally, intermittent dosing regimens have been investigated as a strategy to minimize side effects while maintaining efficacy. For instance, Arriola Apelo et al. (2016) demonstrated that alternative rapamycin treatment schedules could mitigate impacts on glucose homeostasis and the immune system in mice [145]. However, Blagosklonny remained skeptical about the necessity of developing new rapalogs solely to overcome side effects he considered manageable with proper dosing of rapamycin [126, 146]. He emphasized that rapamycin, as a well-studied and FDA-approved drug, could be effectively utilized in anti-aging therapies through personalized dosing strategies without waiting for new drug developments. This perspective underscores the ongoing debate within the geroscience community regarding the optimal approach to mTOR inhibition in aging interventions.

Blagosklonny’s framework for clinical application includes several principles designed to optimize the therapeutic potential of rapamycin:

1. Personalized Dosing: Blagosklonny emphasized individualized dosing regimens, suggesting that optimal results can be achieved through low-dose or intermittent administration tailored to the patient’s tolerance and specific health profile. This approach aims to maximize rapamycin’s benefits while minimizing adverse effects [126, 127, 133, 147–150].
2. Preventive Application: Blagosklonny advocated for the initiation of rapamycin treatment before the onset of age-related diseases, proposing that early intervention could maximize mTOR inhibition’s protective effects against age-associated decline [22, 46, 66, 84, 126, 127, 133, 147, 151–153].
3. Combination Therapy: To further enhance therapeutic outcomes, Blagosklonny proposed combining rapamycin with other agents, such as possibly metformin or ACE inhibitors, that may work synergistically with mTOR inhibition. This approach anticipated the current interest in multi-targeted geroprotective strategies that address multiple aging pathways, reinforcing his forward-looking vision for personalized anti-aging interventions [46, 62, 126, 154]. This approach is gaining traction [101, 142, 155–160].

Blagosklonny’s proposal for “longevity clinics” where patients could receive individualized anti-aging therapies reflects an innovative approach that is garnering significant interest in translational geroscience and longevity medicine [126, 127, 133, 161–170]. These clinics offer a potential framework for Blagosklonny’s vision, where cutting-edge geroprotective treatments can be administered under specialized supervision, translating geroscience insights into practical healthcare solutions.

## CONCLUSION

### Mikhail Blagosklonny’s enduring legacy in geroscience

Dr. Mikhail Blagosklonny’s contributions have fundamentally reshaped geroscience and oncology, offering a pioneering framework for understanding aging not merely as an accumulation of damage but as a quasi-programmed process. His Hyperfunction Theory, centered on the persistent activation of biological growth pathways like mTOR, presents aging as an extension of early-life growth signals that drive cellular and tissue decline over time. By advocating for rapamycin to mitigate these effects, Blagosklonny established a new paradigm that combines theoretical insights with actionable interventions.

Ongoing clinical research into mTOR inhibitors for healthspan and lifespan extension reflects Blagosklonny’s impact, marking a shift in geroscience towards treating aging as a modifiable condition. His vision has inspired a new wave of research focused on interventions that aim not only to extend life but to enhance its quality, underscoring his belief in the potential of translational geroscience.

Blagosklonny’s Hyperfunction Theory offers a compelling alternative to traditional damage-based theories, presenting a novel framework for understanding the causes of aging. This contribution will undoubtedly be remembered in the coming decades and beyond as an innovative contribution to our theoretical grasp of the aging process and a foundation for exploring effective therapeutic approaches. Blagosklonny’s work leaves an enduring legacy, embodying the shift from viewing aging as an inevitable decline to treating it as a condition that science and medicine can manage. As a colleague and friend, Misha’s commitment to advancing geroscience remains a personal and professional inspiration.

## References

[1] Tribute to Dr. Mikhail (Misha) Blagosklonny. Oncotarget. https://www.oncotarget.com/news/pr/tribute-to-dr-mikhail-misha-blagosklonny/.

[2] Peer-reviewed Oncology & Cancer Research Journal. Oncotarget. https://www.oncotarget.com/.

[3] Aging Journal. Aging-US. https://www.aging-us.com.

[4] Cell Cycle. Taylor & Francis. https://www.tandfonline.com/journals/kccy20.

[5] Gladyshev VN. The free radical theory of aging is dead. Long live the damage theory! Antioxid Redox Signal. 2014; 20:727–31. 10.1089/ars.2013.5228 24159899 PMC3901353

[6] de Grey ADN. Programs, Hyperfunction, and Damage: Why Definitions and Logic Matter So Much in Biogerontology. Rejuvenation Res. 2021; 24:83–5. 10.1089/rej.2021.0015 33784821

[7] Lipsky MS, King M. Biological theories of aging. Dis Mon. 2015; 61:460–6. 10.1016/j.disamonth.2015.09.005 26490576

[8] de Grey AD, Ames BN, Andersen JK, Bartke A, Campisi J, Heward CB, McCarter RJ, Stock G. Time to talk SENS: critiquing the immutability of human aging. Ann N Y Acad Sci. 2002; 959:452–62. 10.1111/j.1749-6632.2002.tb02115.x 11976218

[9] Gorbunova V, Seluanov A. Introduction: Progression of the Science of Ageing. Subcell Biochem. 2023; 102:1–6. 10.1007/978-3-031-21410-3_1 36600127

[10] Kennedy BK, Berger SL, Brunet A, Campisi J, Cuervo AM, Epel ES, Franceschi C, Lithgow GJ, Morimoto RI, Pessin JE, Rando TA, Richardson A, Schadt EE, et al. Geroscience: linking aging to chronic disease. Cell. 2014; 159:709–13. 10.1016/j.cell.2014.10.039 25417146 PMC4852871

[11] The Hallmarks of Aging: Cell. https://www.cell.com/cell/fulltext/S0092-8674(13)00645-4.

[12] López-Otín C, Blasco MA, Partridge L, Serrano M, Kroemer G. Hallmarks of aging: An expanding universe. Cell. 2023; 186:243–78. 10.1016/j.cell.2022.11.001 36599349

[13] Franceschi C, Garagnani P, Parini P, Giuliani C, Santoro A. Inflammaging: a new immune-metabolic viewpoint for age-related diseases. Nat Rev Endocrinol. 2018; 14:576–90. 10.1038/s41574-018-0059-4 30046148

[14] Franceschi C, Garagnani P, Vitale G, Capri M, Salvioli S. Inflammaging and 'Garb-aging'. Trends Endocrinol Metab. 2017; 28:199–212. 10.1016/j.tem.2016.09.005 27789101

[15] Niedernhofer LJ, Gurkar AU, Wang Y, Vijg J, Hoeijmakers JHJ, Robbins PD. Nuclear Genomic Instability and Aging. Annu Rev Biochem. 2018; 87:295–322. 10.1146/annurev-biochem-062917-012239 29925262

[16] Loeb LA, Wallace DC, Martin GM. The mitochondrial theory of aging and its relationship to reactive oxygen species damage and somatic mtDNA mutations. Proc Natl Acad Sci U S A. 2005; 102:18769–70. 10.1073/pnas.0509776102 16365283 PMC1323222

[17] Lu YR, Tian X, Sinclair DA. The Information Theory of Aging. Nat Aging. 2023; 3:1486–99. 10.1038/s43587-023-00527-6 38102202

[18] Harman D. Free radical theory of aging: Consequences of mitochondrial aging. AGE. 1983; 6: 86–94. 10.1007/BF02432509

[19] Moorad JA, Promislow DE. A theory of age-dependent mutation and senescence. Genetics. 2008; 179:2061–73. 10.1534/genetics.108.088526 18660535 PMC2516080

[20] Gems D. The hyperfunction theory: An emerging paradigm for the biology of aging. Ageing Res Rev. 2022; 74:101557. 10.1016/j.arr.2021.101557 34990845 PMC7612201

[21] Blagosklonny MV. The hyperfunction theory of aging: three common misconceptions. Oncoscience. 2021; 8:103–7. 10.18632/oncoscience.545 34549076 PMC8448505

[22] Blagosklonny MV. Aging and immortality: quasi-programmed senescence and its pharmacologic inhibition. Cell Cycle. 2006; 5:2087–102. 10.4161/cc.5.18.3288 17012837

[23] Blagosklonny MV. Cellular senescence: when growth stimulation meets cell cycle arrest. Aging (Albany NY). 2023; 15:905–13. 10.18632/aging.204543 36805938 PMC10008486

[24] Blagosklonny MV. Answering the ultimate question "what is the proximal cause of aging?". Aging (Albany NY). 2012; 4:861–77. 10.18632/aging.100525 23425777 PMC3615154

[25] Blagosklonny MV. TOR-driven aging: speeding car without brakes. Cell Cycle. 2009; 8:4055–9. 10.4161/cc.8.24.10310 19923900

[26] Stanfel MN, Shamieh LS, Kaeberlein M, Kennedy BK. The TOR pathway comes of age. Biochim Biophys Acta. 2009; 1790:1067–74. 10.1016/j.bbagen.2009.06.007 19539012 PMC3981532

[27] Blagosklonny MV. Disease or not, aging is easily treatable. Aging (Albany NY). 2018; 10:3067–78. 10.18632/aging.101647 30448823 PMC6286826

[28] Gems D, de la Guardia Y. Alternative Perspectives on Aging in Caenorhabditis elegans: Reactive Oxygen Species or Hyperfunction? Antioxid Redox Signal. 2013; 19:321–9. 10.1089/ars.2012.4840 22870907 PMC5395017

[29] Blagosklonny MV. Aging is not programmed: genetic pseudo-program is a shadow of developmental growth. Cell Cycle. 2013; 12:3736–42. 10.4161/cc.27188 24240128 PMC3905065

[30] Blagosklonny MV. Response to the Thought-Provoking Critique of Hyperfunction Theory by Aubrey de Grey. Rejuvenation Res. 2021; 24:170–2. 10.1089/rej.2021.0018 33784825

[31] Williams: Pleiotropy, natural selection, and the. - Google Scholar. https://scholar.google.com/scholar_lookup?title=Pleiotropy%2C%20natural%20selection%2C%20and%20the%20evolution%20of%20senescence&author=GC.%20Williams&publication_year=1957&journal=Evolution&volume=11&pages=398-411.

[32] Austad SN, Hoffman JM. Is antagonistic pleiotropy ubiquitous in aging biology? Evol Med Public Health. 2018; 2018:287–94. 10.1093/emph/eoy033 30524730 PMC6276058

[33] Blagosklonny MV. Paradoxes of aging. Cell Cycle. 2007; 6:2997–3003. 10.4161/cc.6.24.5124 18156807

[34] Genetics of Longevity in Model Organisms: Debates and Paradigm Shifts. Annual Reviews. https://www.annualreviews.org/content/journals/10.1146/annurev-physiol-030212-183712. 10.1146/annurev-physiol-030212-183712 23190075

[35] de la Guardia Y, Gilliat AF, Hellberg J, Rennert P, Cabreiro F, Gems D. Run-on of germline apoptosis promotes gonad senescence in C. elegans. Oncotarget. 2016; 7:39082–96. 10.18632/oncotarget.9681 27256978 PMC5129915

[36] Blagosklonny MV. Molecular damage in cancer: an argument for mTOR-driven aging. Aging (Albany NY). 2011; 3:1130–41. 10.18632/aging.100422 22246147 PMC3273893

[37] de Magalhães JP. Programmatic features of aging originating in development: aging mechanisms beyond molecular damage? FASEB J. 2012; 26:4821–6. 10.1096/fj.12-210872 22964300 PMC3509060

[38] de Magalhães JP, Church GM. Genomes optimize reproduction: aging as a consequence of the developmental program. Physiology (Bethesda). 2005; 20:252–9. 10.1152/physiol.00010.2005 16024513

[39] Gems D, Kern CC. Biological constraint, evolutionary spandrels and antagonistic pleiotropy. Ageing Res Rev. 2024; 101:102527. 10.1016/j.arr.2024.102527 39374830 PMC7618566

[40] Slade L, Etheridge T, Szewczyk NJ. Consolidating multiple evolutionary theories of ageing suggests a need for new approaches to study genetic contributions to ageing decline. Ageing Res Rev. 2024; 100:102456. 10.1016/j.arr.2024.102456 39153601

[41] Lemaître JF, Moorad J, Gaillard JM, Maklakov AA, Nussey DH. A unified framework for evolutionary genetic and physiological theories of aging. PLoS Biol. 2024; 22:e3002513. 10.1371/journal.pbio.3002513 38412150 PMC10898761

[42] Maklakov AA, Chapman T. Evolution of ageing as a tangle of trade-offs: energy versus function. Proc Biol Sci. 2019; 286:20191604. 10.1098/rspb.2019.1604 31530150 PMC6784717

[43] Galimov ER, Lohr JN, Gems D. When and How Can Death Be an Adaptation? Biochemistry (Mosc). 2019; 84:1433–7. 10.1134/S0006297919120010 31870246

[44] Lohr JN, Galimov ER, Gems D. Does senescence promote fitness in Caenorhabditis elegans by causing death? Ageing Res Rev. 2019; 50:58–71. 10.1016/j.arr.2019.01.008 30639341 PMC6520499

[45] Blagosklonny MV. Cell senescence and hypermitogenic arrest. EMBO Rep. 2003; 4:358–62. 10.1038/sj.embor.embor806 12671679 PMC1319162

[46] Blagosklonny MV. An anti-aging drug today: from senescence-promoting genes to anti-aging pill. Drug Discov Today. 2007; 12:218–24. 10.1016/j.drudis.2007.01.004 17331886

[47] Blagosklonny MV. Prospective treatment of age-related diseases by slowing down aging. Am J Pathol. 2012; 181:1142–6. 10.1016/j.ajpath.2012.06.024 22841821

[48] Blagosklonny MV. Rejuvenating immunity: "anti-aging drug today" eight years later. Oncotarget. 2015; 6:19405–12. 10.18632/oncotarget.3740 25844603 PMC4637294

[49] Lages CS, Lewkowich I, Sproles A, Wills-Karp M, Chougnet C. Partial restoration of T-cell function in aged mice by in vitro blockade of the PD-1/ PD-L1 pathway. Aging Cell. 2010; 9:785–98. 10.1111/j.1474-9726.2010.00611.x 20653631 PMC2941565

[50] Hurez V, Dao V, Liu A, Pandeswara S, Gelfond J, Sun L, Bergman M, Orihuela CJ, Galvan V, Padrón Á, Drerup J, Liu Y, Hasty P, et al. Chronic mTOR inhibition in mice with rapamycin alters T, B, myeloid, and innate lymphoid cells and gut flora and prolongs life of immune-deficient mice. Aging Cell. 2015; 14:945–56. 10.1111/acel.12380 26315673 PMC4693453

[51] Diken M, Kreiter S, Vascotto F, Selmi A, Attig S, Diekmann J, Huber C, Türeci Ö, Sahin U. mTOR inhibition improves antitumor effects of vaccination with antigen-encoding RNA. Cancer Immunol Res. 2013; 1:386–92. 10.1158/2326-6066.CIR-13-0046 24778131

[52] Keating R, Hertz T, Wehenkel M, Harris TL, Edwards BA, McClaren JL, Brown SA, Surman S, Wilson ZS, Bradley P, Hurwitz J, Chi H, Doherty PC, et al. The kinase mTOR modulates the antibody response to provide cross-protective immunity to lethal infection with influenza virus. Nat Immunol. 2013; 14:1266–76. 10.1038/ni.2741 24141387 PMC3883080

[53] Mannick JB, Del Giudice G, Lattanzi M, Valiante NM, Praestgaard J, Huang B, Lonetto MA, Maecker HT, Kovarik J, Carson S, Glass DJ, Klickstein LB. mTOR inhibition improves immune function in the elderly. Sci Transl Med. 2014; 6:268ra179. 10.1126/scitranslmed.3009892 25540326

[54] Mannick JB, Morris M, Hockey HP, Roma G, Beibel M, Kulmatycki K, Watkins M, Shavlakadze T, Zhou W, Quinn D, Glass DJ, Klickstein LB. TORC1 inhibition enhances immune function and reduces infections in the elderly. Sci Transl Med. 2018; 10:eaaq1564. 10.1126/scitranslmed.aaq1564 29997249

[55] Mannick JB, Teo G, Bernardo P, Quinn D, Russell K, Klickstein L, Marshall W, Shergill S. Targeting the biology of ageing with mTOR inhibitors to improve immune function in older adults: phase 2b and phase 3 randomised trials. Lancet Healthy Longev. 2021; 2:e250–62. 10.1016/S2666-7568(21)00062-3 33977284 PMC8102040

[56] Chen C, Liu Y, Liu Y, Zheng P. mTOR regulation and therapeutic rejuvenation of aging hematopoietic stem cells. Sci Signal. 2009; 2:ra75. 10.1126/scisignal.2000559 19934433 PMC4020596

[57] Ando S, Perkins CM, Sajiki Y, Chastain C, Valanparambil RM, Wieland A, Hudson WH, Hashimoto M, Ramalingam SS, Freeman GJ, Ahmed R, Araki K. mTOR regulates T cell exhaustion and PD-1-targeted immunotherapy response during chronic viral infection. J Clin Invest. 2023; 133:e160025. 10.1172/JCI160025 36378537 PMC9843061

[58] Rolland Y, Sierra F, Ferrucci L, Barzilai N, De Cabo R, Mannick J, Oliva A, Evans W, Angioni D, De Souto Barreto P, Raffin J, Vellas B, Kirkland JL, and G. C.T-TF group. Challenges in developing Geroscience trials. Nat Commun. 2023; 14:5038. 10.1038/s41467-023-39786-7 37598227 PMC10439920

[59] Dai DF, Karunadharma PP, Chiao YA, Basisty N, Crispin D, Hsieh EJ, Chen T, Gu H, Djukovic D, Raftery D, Beyer RP, MacCoss MJ, Rabinovitch PS. Altered proteome turnover and remodeling by short-term caloric restriction or rapamycin rejuvenate the aging heart. Aging Cell. 2014; 13:529–39. 10.1111/acel.12203 24612461 PMC4040127

[60] Flynn JM, O'Leary MN, Zambataro CA, Academia EC, Presley MP, Garrett BJ, Zykovich A, Mooney SD, Strong R, Rosen CJ, Kapahi P, Nelson MD, Kennedy BK, Melov S. Late-life rapamycin treatment reverses age-related heart dysfunction. Aging Cell. 2013; 12:851–62. 10.1111/acel.12109 23734717 PMC4098908

[61] Gao G, Chen W, Yan M, Liu J, Luo H, Wang C, Yang P. Rapamycin regulates the balance between cardiomyocyte apoptosis and autophagy in chronic heart failure by inhibiting mTOR signaling. Int J Mol Med. 2020; 45:195–209. 10.3892/ijmm.2019.4407 31746373 PMC6889932

[62] Blagosklonny MV. From rapalogs to anti-aging formula. Oncotarget. 2017; 8:35492–507. 10.18632/oncotarget.18033 28548953 PMC5482593

[63] Urfer SR, Kaeberlein TL, Mailheau S, Bergman PJ, Creevy KE, Promislow DEL, Kaeberlein M. A randomized controlled trial to establish effects of short-term rapamycin treatment in 24 middle-aged companion dogs. Geroscience. 2017; 39:117–27. 10.1007/s11357-017-9972-z 28374166 PMC5411365

[64] Barnett BG, Wesselowski SR, Gordon SG, Saunders AB, Promislow DEL, Schwartz SM, Chou L, Evans JB, Kaeberlein M, Creevy KE. A masked, placebo-controlled, randomized clinical trial evaluating safety and the effect on cardiac function of low-dose rapamycin in 17 healthy client-owned dogs. Front Vet Sci. 2023; 10. https://www.frontiersin.org/journals/veterinary-science/articles/10.3389/fvets.2023.1168711/full. 10.3389/fvets.2023.1168711 PMC10233048 37275618

[65] North BJ, Sinclair DA. The intersection between aging and cardiovascular disease. Circ Res. 2012; 110:1097–108. 10.1161/CIRCRESAHA.111.246876 22499900 PMC3366686

[66] Blagosklonny: Solving puzzles of aging: From disposable. - Google Scholar. https://scholar.google.com/scholar?q=related:fqgc2HDY1qwJ:scholar.google.com/&scioq=blagosklonny+dementia&hl=en&as_sdt=0,7.

[67] Li X, McPherson M, Hager M, Lee M, Chang P, Miller RA. Four anti-aging drugs and calorie-restricted diet produce parallel effects in fat, brain, muscle, macrophages, and plasma of young mice. Geroscience. 2023; 45:2495–510. 10.1007/s11357-023-00770-0 36920743 PMC10651632

[68] An WL, Cowburn RF, Li L, Braak H, Alafuzoff I, Iqbal K, Iqbal IG, Winblad B, Pei JJ. Up-regulation of phosphorylated/activated p70 S6 kinase and its relationship to neurofibrillary pathology in Alzheimer's disease. Am J Pathol. 2003; 163:591–607. 10.1016/S0002-9440(10)63687-5 12875979 PMC1868198

[69] Caccamo A, Majumder S, Richardson A, Strong R, Oddo S. Molecular interplay between mammalian target of rapamycin (mTOR), amyloid-beta, and Tau: effects on cognitive impairments. J Biol Chem. 2010; 285:13107–20. 10.1074/jbc.M110.100420 20178983 PMC2857107

[70] Van Skike CE, Lin AL, Roberts Burbank R, Halloran JJ, Hernandez SF, Cuvillier J, Soto VY, Hussong SA, Jahrling JB, Javors MA, Hart MJ, Fischer KE, Austad SN, Galvan V. mTOR drives cerebrovascular, synaptic, and cognitive dysfunction in normative aging. Aging Cell. 2020; 19:e13057. 10.1111/acel.13057 31693798 PMC6974719

[71] Hou SJ, Zhang SX, Li Y, Xu SY. Rapamycin Responds to Alzheimer's Disease: A Potential Translational Therapy. Clin Interv Aging. 2023; 18:1629–39. 10.2147/CIA.S429440 37810956 PMC10557994

[72] Kaeberlein M, Galvan V. Rapamycin and Alzheimer's disease: Time for a clinical trial? Sci Transl Med. 2019; 11:eaar4289. 10.1126/scitranslmed.aar4289 30674654 PMC6762017

[73] Carosi JM, Sargeant TJ. Rapamycin and Alzheimer disease: a double-edged sword? Autophagy. 2019; 15:1460–2. 10.1080/15548627.2019.1615823 31066320 PMC6613906

[74] Whyte LS, Lau AA, Hemsley KM, Hopwood JJ, Sargeant TJ. Endo-lysosomal and autophagic dysfunction: a driving factor in Alzheimer's disease? J Neurochem. 2017; 140:703–17. 10.1111/jnc.13935 28027395

[75] Richardson A, Galvan V, Lin AL, Oddo S. How longevity research can lead to therapies for Alzheimer's disease: The rapamycin story. Exp Gerontol. 2015; 68:51–8. 10.1016/j.exger.2014.12.002 25481271 PMC6417920

[76] Khan MR, Yin X, Kang SU, Mitra J, Wang H, Ryu T, Brahmachari S, Karuppagounder SS, Kimura Y, Jhaldiyal A, Kim HH, Gu H, Chen R, et al. Enhanced mTORC1 signaling and protein synthesis in pathologic α-synuclein cellular and animal models of Parkinson's disease. Sci Transl Med. 2023; 15:eadd0499. 10.1126/scitranslmed.add0499 38019930

[77] King MA, Hands S, Hafiz F, Mizushima N, Tolkovsky AM, Wyttenbach A. Rapamycin inhibits polyglutamine aggregation independently of autophagy by reducing protein synthesis. Mol Pharmacol. 2008; 73:1052–63. 10.1124/mol.107.043398 18199701

[78] Blagosklonny MV. Selective anti-cancer agents as anti-aging drugs. Cancer Biol Ther. 2013; 14:1092–7. 10.4161/cbt.27350 24345884 PMC3912031

[79] Blagosklonny MV. Common drugs and treatments for cancer and age-related diseases: revitalizing answers to NCI's provocative questions. Oncotarget. 2012; 3:1711–24. 10.18632/oncotarget.890 23565531 PMC3681506

[80] Leontieva OV, Novototskaya LR, Paszkiewicz GM, Komarova EA, Gudkov AV, Blagosklonny MV. Dysregulation of the mTOR pathway in p53-deficient mice. Cancer Biol Ther. 2013; 14:1182–8. 10.4161/cbt.26947 24184801 PMC3912042

[81] Blagosklonny MV. Geroconversion: irreversible step to cellular senescence. Cell Cycle. 2014; 13:3628–35. 10.4161/15384101.2014.985507 25483060 PMC4614001

[82] Blagosklonny MV. Cell cycle arrest is not senescence. Aging (Albany NY). 2011; 3:94–101. 10.18632/aging.100281 21297220 PMC3082019

[83] Leontieva OV, Demidenko ZN, Gudkov AV, Blagosklonny MV. Elimination of proliferating cells unmasks the shift from senescence to quiescence caused by rapamycin. PLoS One. 2011; 6:e26126. 10.1371/journal.pone.0026126 22022534 PMC3191182

[84] Blagosklonny MV. Prevention of cancer by inhibiting aging. Cancer Biol Ther. 2008; 7:1520–4. 10.4161/cbt.7.10.6663 18769112

[85] Anisimov VN, Zabezhinski MA, Popovich IG, Piskunova TS, Semenchenko AV, Tyndyk ML, Yurova MN, Antoch MP, Blagosklonny MV. Rapamycin extends maximal lifespan in cancer-prone mice. Am J Pathol. 2010; 176:2092–7. 10.2353/ajpath.2010.091050 20363920 PMC2861075

[86] Antoch MP, Wrobel M, Gillard B, Kuropatwinski KK, Toshkov I, Gleiberman AS, Karasik E, Moser MT, Foster BA, Andrianova EL, Chernova OV, Gudkov AV. Superior cancer preventive efficacy of low versus high dose of mTOR inhibitor in a mouse model of prostate cancer. Oncotarget. 2020; 11:1373–87. 10.18632/oncotarget.27550 32341756 PMC7170500

[87] Anisimov VN, Zabezhinski MA, Popovich IG, Piskunova TS, Semenchenko AV, Tyndyk ML, Yurova MN, Rosenfeld SV, Blagosklonny MV. Rapamycin increases lifespan and inhibits spontaneous tumorigenesis in inbred female mice. Cell Cycle. 2011; 10:4230–6. 10.4161/cc.10.24.18486 22107964

[88] Komarova EA, Antoch MP, Novototskaya LR, Chernova OB, Paszkiewicz G, Leontieva OV, Blagosklonny MV, Gudkov AV. Rapamycin extends lifespan and delays tumorigenesis in heterozygous p53+/- mice. Aging (Albany NY). 2012; 4:709–14. 10.18632/aging.100498 23123616 PMC3517941

[89] Van Meter M, Seluanov A, Gorbunova V. Forever young? Exploring the link between rapamycin, longevity and cancer. Cell Cycle. 2012; 11:4296–7. 10.4161/cc.22868 23152185 PMC3552905

[90] Finkel T, Serrano M, Blasco MA. The common biology of cancer and ageing. Nature. 2007; 448:767–74. 10.1038/nature05985 17700693

[91] Cornu M, Albert V, Hall MN. mTOR in aging, metabolism, and cancer. Curr Opin Genet Dev. 2013; 23:53–62. 10.1016/j.gde.2012.12.005 23317514

[92] Khanna A, Kapahi P. Rapamycin: killing two birds with one stone. Aging (Albany NY). 2011; 3:1043–4. 10.18632/aging.100405 22170738 PMC3249449

[93] Zhang A, Meecham-Garcia G, Nguyen Hong C, Xie P, Kern CC, Zhang B, Chapman H, Gems D. Characterization of Effects of mTOR Inhibitors on Aging in Caenorhabditis elegans. J Gerontol A Biol Sci Med Sci. 2024; 79:glae196. 10.1093/gerona/glae196 39150882 PMC11374883

[94] Bjedov I, Toivonen JM, Kerr F, Slack C, Jacobson J, Foley A, Partridge L. Mechanisms of life span extension by rapamycin in the fruit fly Drosophila melanogaster. Cell Metab. 2010; 11:35–46. 10.1016/j.cmet.2009.11.010 20074526 PMC2824086

[95] Harrison DE, Strong R, Sharp ZD, Nelson JF, Astle CM, Flurkey K, Nadon NL, Wilkinson JE, Frenkel K, Carter CS, Pahor M, Javors MA, Fernandez E, Miller RA. Rapamycin fed late in life extends lifespan in genetically heterogeneous mice. Nature. 2009; 460:392–5. 10.1038/nature08221 19587680 PMC2786175

[96] Arriola Apelo SI, Pumper CP, Baar EL, Cummings NE, Lamming DW. Intermittent Administration of Rapamycin Extends the Life Span of Female C57BL/6J Mice. J Gerontol A Biol Sci Med Sci. 2016; 71:876–81. 10.1093/gerona/glw064 27091134 PMC4906329

[97] Bitto A, Ito TK, Pineda VV, LeTexier NJ, Huang HZ, Sutlief E, Tung H, Vizzini N, Chen B, Smith K, Meza D, Yajima M, Beyer RP, et al. Transient rapamycin treatment can increase lifespan and healthspan in middle-aged mice. Elife. 2016; 5:e16351. 10.7554/eLife.16351 27549339 PMC4996648

[98] Leontieva OV, Paszkiewicz GM, Blagosklonny MV. Weekly administration of rapamycin improves survival and biomarkers in obese male mice on high-fat diet. Aging Cell. 2014; 13:616–22. 10.1111/acel.12211 24655348 PMC4326934

[99] Miller RA, Harrison DE, Astle CM, Fernandez E, Flurkey K, Han M, Javors MA, Li X, Nadon NL, Nelson JF, Pletcher S, Salmon AB, Sharp ZD, et al. Rapamycin-mediated lifespan increase in mice is dose and sex dependent and metabolically distinct from dietary restriction. Aging Cell. 2014; 13:468–77. 10.1111/acel.12194 24341993 PMC4032600

[100] Miller RA, Harrison DE, Astle CM, Baur JA, Boyd AR, de Cabo R, Fernandez E, Flurkey K, Javors MA, Nelson JF, Orihuela CJ, Pletcher S, Sharp ZD, et al. Rapamycin, but not resveratrol or simvastatin, extends life span of genetically heterogeneous mice. J Gerontol A Biol Sci Med Sci. 2011; 66:191–201. 10.1093/gerona/glq178 20974732 PMC3021372

[101] Strong R, Miller RA, Cheng CJ, Nelson JF, Gelfond J, Allani SK, Diaz V, Dorigatti AO, Dorigatti J, Fernandez E, Galecki A, Ginsburg B, Hamilton KL, et al. Lifespan benefits for the combination of rapamycin plus acarbose and for captopril in genetically heterogeneous mice. Aging Cell. 2022; 21:e13724. 10.1111/acel.13724 36179270 PMC9741502

[102] Wilkinson JE, Burmeister L, Brooks SV, Chan CC, Friedline S, Harrison DE, Hejtmancik JF, Nadon N, Strong R, Wood LK, Woodward MA, Miller RA. Rapamycin slows aging in mice. Aging Cell. 2012; 11:675–82. 10.1111/j.1474-9726.2012.00832.x 22587563 PMC3434687

[103] Zhang Y, Bokov A, Gelfond J, Soto V, Ikeno Y, Hubbard G, Diaz V, Sloane L, Maslin K, Treaster S, Réndon S, van Remmen H, Ward W, et al. Rapamycin extends life and health in C57BL/6 mice. J Gerontol A Biol Sci Med Sci. 2014; 69:119–30. 10.1093/gerona/glt056 23682161 PMC4038246

[104] Swindell WR. Meta-Analysis of 29 Experiments Evaluating the Effects of Rapamycin on Life Span in the Laboratory Mouse. J Gerontol A Biol Sci Med Sci. 2017; 72:1024–32. 10.1093/gerona/glw153 27519886

[105] Selvarani R, Mohammed S, Richardson A. Effect of rapamycin on aging and age-related diseases-past and future. Geroscience. 2021; 43:1135–58. 10.1007/s11357-020-00274-1 33037985 PMC8190242

[106] Shindyapina AV, Cho Y, Kaya A, Tyshkovskiy A, Castro JP, Deik A, Gordevicius J, Poganik JR, Clish CB, Horvath S, Peshkin L, Gladyshev VN. Rapamycin treatment during development extends life span and health span of male mice and *Daphnia magna*. Sci Adv. 2022; 8:eabo5482. 10.1126/sciadv.abo5482 36112674 PMC9481125

[107] Chung CL, Lawrence I, Hoffman M, Elgindi D, Nadhan K, Potnis M, Jin A, Sershon C, Binnebose R, Lorenzini A, Sell C. Topical rapamycin reduces markers of senescence and aging in human skin: an exploratory, prospective, randomized trial. Geroscience. 2019; 41:861–9. 10.1007/s11357-019-00113-y 31761958 PMC6925069

[108] Dou X, Sun Y, Li J, Zhang J, Hao D, Liu W, Wu R, Kong F, Peng X, Li J. Short-term rapamycin treatment increases ovarian lifespan in young and middle-aged female mice. Aging Cell. 2017; 16:825–36. 10.1111/acel.12617 28544226 PMC5506398

[109] Garcia DN, Saccon TD, Pradiee J, Rincón JAA, Andrade KRS, Rovani MT, Mondadori RG, Cruz LAX, Barros CC, Masternak MM, Bartke A, Mason JB, Schneider A. Effect of caloric restriction and rapamycin on ovarian aging in mice. Geroscience. 2019; 41:395–408. 10.1007/s11357-019-00087-x 31359237 PMC6815295

[110] Hine C. Rapamycin keeps the reproductive clock ticking. Sci Transl Med. 2017; 9:eaan4296. 10.1126/scitranslmed.aan4296 28566425

[111] Blagosklonny MV. Rapamycin extends life-and health span because it slows aging. Aging (Albany NY). 2013; 5:592–8. 10.18632/aging.100591 23934728 PMC3796212

[112] Hudson J, Kaeberlein T, Mahal A, Wong N, Ghorbanifarajzadeh M, Radella F, Isman A, Nyquist A, Zalzala S, Haddad G, Kaeberlein M, An JY. Evaluation of off-label rapamycin use on oral health. Geroscience. 2024; 46:4135–46. 10.1007/s11357-024-01221-0 38839644 PMC11335702

[113] Weintraub JA, Kaeberlein M, Perissinotto C, Atchison KA, Chen X, D'Souza RN, Feine JS, Ghezzi EM, Kirkwood KL, Ryder M, Slashcheva LD, Touger-Decker R, Wu B, Kapila Y. Geroscience: Aging and Oral Health Research. Adv Dent Res. 2023; 31:2–15. 10.1177/08959374231200840 37933846 PMC10767691

[114] Ye L, Widlund AL, Sims CA, Lamming DW, Guan Y, Davis JG, Sabatini DM, Harrison DE, Vang O, Baur JA. Rapamycin doses sufficient to extend lifespan do not compromise muscle mitochondrial content or endurance. Aging (Albany NY). 2013; 5:539–50. 10.18632/aging.100576 23929887 PMC3765582

[115] Markofski MM, Dickinson JM, Drummond MJ, Fry CS, Fujita S, Gundermann DM, Glynn EL, Jennings K, Paddon-Jones D, Reidy PT, Sheffield-Moore M, Timmerman KL, Rasmussen BB, Volpi E. Effect of age on basal muscle protein synthesis and mTORC1 signaling in a large cohort of young and older men and women. Exp Gerontol. 2015; 65:1–7. 10.1016/j.exger.2015.02.015 25735236 PMC4397165

[116] Joseph GA, Wang SX, Jacobs CE, Zhou W, Kimble GC, Tse HW, Eash JK, Shavlakadze T, Glass DJ. Partial Inhibition of mTORC1 in Aged Rats Counteracts the Decline in Muscle Mass and Reverses Molecular Signaling Associated with Sarcopenia. Mol Cell Biol. 2019; 39:e00141–19. 10.1128/MCB.00141-19 31308131 PMC6751631

[117] White Z, White RB, McMahon C, Grounds MD, Shavlakadze T. High mTORC1 signaling is maintained, while protein degradation pathways are perturbed in old murine skeletal muscles in the fasted state. Int J Biochem Cell Biol. 2016; 78:10–21. 10.1016/j.biocel.2016.06.012 27343428

[118] Arriola Apelo SI, Lamming DW. Rapamycin: An InhibiTOR of Aging Emerges From the Soil of Easter Island. J Gerontol A Biol Sci Med Sci. 2016; 71:841–9. 10.1093/gerona/glw090 27208895 PMC4906330

[119] Lamming DW, Ye L, Sabatini DM, Baur JA. Rapalogs and mTOR inhibitors as anti-aging therapeutics. J Clin Invest. 2013; 123:980–9. 10.1172/JCI64099 23454761 PMC3582126

[120] Konopka AR, Lamming DW, and RAP PAC Investigators EVERLAST Investigators. Blazing a trail for the clinical use of rapamycin as a geroprotecTOR. Geroscience. 2023; 45:2769–83. 10.1007/s11357-023-00935-x 37801202 PMC10643772

[121] Lamming DW, Salmon AB. TORwards a Victory Over Aging. J Gerontol A Biol Sci Med Sci. 2020; 75:1–3. 10.1093/gerona/glz212 31544928 PMC7328347

[122] Neff F, Flores-Dominguez D, Ryan DP, Horsch M, Schröder S, Adler T, Afonso LC, Aguilar-Pimentel JA, Becker L, Garrett L, Hans W, Hettich MM, Holtmeier R, et al. Rapamycin extends murine lifespan but has limited effects on aging. J Clin Invest. 2013; 123:3272–91. 10.1172/JCI67674 23863708 PMC3726163

[123] Zaseck LW, Miller RA, Brooks SV. Rapamycin Attenuates Age-associated Changes in Tibialis Anterior Tendon Viscoelastic Properties. J Gerontol A Biol Sci Med Sci. 2016; 71:858–65. 10.1093/gerona/glv307 26809496 PMC4906327

[124] Liao CY, Anderson SS, Chicoine NH, Mayfield JR, Academia EC, Wilson JA, Pongkietisak C, Thompson MA, Lagmay EP, Miller DM, Hsu YM, McCormick MA, O'Leary MN, Kennedy BK. Rapamycin Reverses Metabolic Deficits in Lamin A/C-Deficient Mice. Cell Rep. 2016; 17:2542–52. 10.1016/j.celrep.2016.10.040 27926859 PMC6594831

[125] Zhang Y, Zhang J, Wang S. The Role of Rapamycin in Healthspan Extension via the Delay of Organ Aging. Ageing Res Rev. 2021; 70:101376. 10.1016/j.arr.2021.101376 34089901

[126] Blagosklonny MV. Rapamycin for longevity: opinion article. Aging (Albany NY). 2019; 11:8048–67. 10.18632/aging.102355 31586989 PMC6814615

[127] Blagosklonny MV. Towards disease-oriented dosing of rapamycin for longevity: does aging exist or only age-related diseases? Aging (Albany NY). 2023; 15:6632–40. 10.18632/aging.204920 37477535 PMC10415559

[128] Strong R, Miller RA, Bogue M, Fernandez E, Javors MA, Libert S, Marinez PA, Murphy MP, Musi N, Nelson JF, Petrascheck M, Reifsnyder P, Richardson A, et al. Rapamycin-mediated mouse lifespan extension: Late-life dosage regimes with sex-specific effects. Aging Cell. 2020; 19:e13269. 10.1111/acel.13269 33145977 PMC7681050

[129] Sierra F. Moving Geroscience Into Uncharted Waters. J Gerontol A Biol Sci Med Sci. 2016; 71:1385–7. 10.1093/gerona/glw087 27535965

[130] Kaeberlein M. Translational geroscience: A new paradigm for 21st century medicine. Transl Med Aging. 2017; 1:1–4. 10.1016/j.tma.2017.09.004 32219192 PMC7098696

[131] Lee MB, Kaeberlein M. Translational Geroscience: From invertebrate models to companion animal and human interventions. Transl Med Aging. 2018; 2:15–29. 10.1016/j.tma.2018.08.002 32368707 PMC7198054

[132] Austad SN. The Geroscience Hypothesis: Is It Possible to Change the Rate of Aging? In: Sierra F, Kohanski R, editors. Advances in Geroscience. Cham: Springer International Publishing. 2016; 1–36. 10.1007/978-3-319-23246-1_1

[133] Blagosklonny MV. The goal of geroscience is life extension. Oncotarget. 2021; 12:131–44. 10.18632/oncotarget.27882 33613842 PMC7869575

[134] Kaeberlein TL, Green AS, Haddad G, Hudson J, Isman A, Nyquist A, Rosen BS, Suh Y, Zalzala S, Zhang X, Blagosklonny MV, An JY, Kaeberlein M. Evaluation of off-label rapamycin use to promote healthspan in 333 adults. Geroscience. 2023; 45:2757–68. 10.1007/s11357-023-00818-1 37191826 PMC10187519

[135] Sierra F, Caspi A, Fortinsky RH, Haynes L, Lithgow GJ, Moffitt TE, Olshansky SJ, Perry D, Verdin E, Kuchel GA. Moving geroscience from the bench to clinical care and health policy. J Am Geriatr Soc. 2021; 69:2455–63. 10.1111/jgs.17301 34145908 PMC10202082

[136] Harinath G, Lee V, Nyquist A, Moel M, Hagemeier J, Morgan SL, Isman A, Zalzala S. Safety and efficacy of rapamycin on healthspan metrics after one year: PEARL Trial Results. medRxiv. 2024. https://www.medrxiv.org/content/10.1101/2024.08.21.24312372v1. 10.18632/aging.206235 PMC12074816 40188830

[137] Stanfield B, Kaeberlein M, Leroux B, Jones J, Lucas R, Arroll B. A single-center, double-blind, randomized, placebo-controlled, two-arm study to evaluate the safety and efficacy of once-weekly sirolimus (rapamycin) on muscle strength and endurance in older adults following a 13-week exercise program. Trials. 2024; 25:642. 10.1186/s13063-024-08490-2 39354527 PMC11443903

[138] Longo VD, Antebi A, Bartke A, Barzilai N, Brown-Borg HM, Caruso C, Curiel TJ, de Cabo R, Franceschi C, Gems D, Ingram DK, Johnson TE, Kennedy BK, et al. Interventions to Slow Aging in Humans: Are We Ready? Aging Cell. 2015; 14:497–510. 10.1111/acel.12338 25902704 PMC4531065

[139] Barzilai N, Cuervo AM, Austad S. Aging as a Biological Target for Prevention and Therapy. JAMA. 2018; 320:1321–2. 10.1001/jama.2018.9562 30242337

[140] Cox LS. Live fast, die young: new lessons in mammalian longevity. Rejuvenation Res. 2009; 12:283–8. 10.1089/rej.2009.0894 19725776

[141] Baghdadi M, Nespital T, Monzó C, Deelen J, Grönke S, Partridge L. Intermittent rapamycin feeding recapitulates some effects of continuous treatment while maintaining lifespan extension. Mol Metab. 2024; 81:101902. 10.1016/j.molmet.2024.101902 38360109 PMC10900781

[142] Gkioni L, Nespital T, Monzó C, Bali J, Nassr T, Cremer AL, Beyer A, Backes H, Grönke S, Partridge L. A combination of the geroprotectors trametinib and rapamycin is more effective than either drug alone. bioRxiv. 2024. https://www.biorxiv.org/content/10.1101/2024.07.25.605097v1. 10.1038/s43587-025-00876-4 PMC12270913 40437307

[143] Campisi J, Kapahi P, Lithgow GJ, Melov S, Newman JC, Verdin E. From discoveries in ageing research to therapeutics for healthy ageing. Nature. 2019; 571:183–92. 10.1038/s41586-019-1365-2 31292558 PMC7205183

[144] Moskalev A, Chernyagina E, Tsvetkov V, Fedintsev A, Shaposhnikov M, Krut'ko V, Zhavoronkov A, Kennedy BK. Developing criteria for evaluation of geroprotectors as a key stage toward translation to the clinic. Aging Cell. 2016; 15:407–15. 10.1111/acel.12463 26970234 PMC4854916

[145] Arriola Apelo SI, Neuman JC, Baar EL, Syed FA, Cummings NE, Brar HK, Pumper CP, Kimple ME, Lamming DW. Alternative rapamycin treatment regimens mitigate the impact of rapamycin on glucose homeostasis and the immune system. Aging Cell. 2016; 15:28–38. 10.1111/acel.12405 26463117 PMC4717280

[146] Blagosklonny MV. Fasting and rapamycin: diabetes versus benevolent glucose intolerance. Cell Death Dis. 2019; 10:607. 10.1038/s41419-019-1822-8 31406105 PMC6690951

[147] Blagosklonny MV. Why human lifespan is rapidly increasing: solving "longevity riddle" with "revealed-slow-aging" hypothesis. Aging (Albany NY). 2010; 2:177–82. 10.18632/aging.100139 20404395 PMC2881507

[148] Blagosklonny MV. How to save Medicare: the anti-aging remedy. Aging (Albany NY). 2012; 4:547–52. 10.18632/aging.100479 22915707 PMC3461342

[149] Gonzalez-Freire M, Diaz-Ruiz A, Hauser D, Martinez-Romero J, Ferrucci L, Bernier M, de Cabo R. The road ahead for health and lifespan interventions. Ageing Res Rev. 2020; 59:101037. 10.1016/j.arr.2020.101037 32109604 PMC7450388

[150] Moskalev A, Anisimov V, Aliper A, Artemov A, Asadullah K, Belsky D, Baranova A, de Grey A, Dixit VD, Debonneuil E, Dobrovolskaya E, Fedichev P, Fedintsev A, et al. A review of the biomedical innovations for healthy longevity. Aging (Albany NY). 2017; 9:7–25. 10.18632/aging.101163

[151] Blagosklonny MV. Are menopause, aging and prostate cancer diseases? Aging (Albany NY). 2023; 15:298–307. 10.18632/aging.204499 36707068 PMC9925691

[152] Blagosklonny MV. No limit to maximal lifespan in humans: how to beat a 122-year-old record. Oncoscience. 2021; 8:110–9. 10.18632/oncoscience.547 34869788 PMC8636159

[153] Longo VD. Programmed longevity, youthspan, and juventology. Aging Cell. 2019; 18:e12843. 10.1111/acel.12843 30334314 PMC6351819

[154] Blagosklonny MV. Koschei the immortal and anti-aging drugs. Cell Death Dis. 2014; 5:e1552. 10.1038/cddis.2014.520 25476900 PMC4649836

[155] Lewis CJ, de Grey AD. Combining rejuvenation interventions in rodents: a milestone in biomedical gerontology whose time has come. Expert Opin Ther Targets. 2024; 28:501–11. 10.1080/14728222.2024.2330425 38477630

[156] Nespital T, Neuhaus B, Mesaros A, Pahl A, Partridge L. Lithium can mildly increase health during ageing but not lifespan in mice. Aging Cell. 2021; 20:e13479. 10.1111/acel.13479 34532960 PMC8520709

[157] Pitt JN, Strait NL, Vayndorf EM, Blue BW, Tran CH, Davis BEM, Huang K, Johnson BJ, Lim KM, Liu S, Nikjoo A, Vaid A, Wu JZ, Kaeberlein M. WormBot, an open-source robotics platform for survival and behavior analysis in C. elegans. Geroscience. 2019; 41:961–73. 10.1007/s11357-019-00124-9 31728898 PMC6925079

[158] Lee MB, Blue B, Muir M, Kaeberlein M. The million-molecule challenge: a moonshot project to rapidly advance longevity intervention discovery. Geroscience. 2023; 45:3103–13. 10.1007/s11357-023-00867-6 37432607 PMC10643437

[159] ITP. National Institute on Aging. https://www.nia.nih.gov/research/dab/interventions-testing-program-itp.

[160] Castillo-Quan JI, Tain LS, Kinghorn KJ, Li L, Grönke S, Hinze Y, Blackwell TK, Bjedov I, Partridge L. A triple drug combination targeting components of the nutrient-sensing network maximizes longevity. Proc Natl Acad Sci U S A. 2019; 116:20817–9. 10.1073/pnas.1913212116 31570569 PMC6800352

[161] Bischof E, Scheibye-Knudsen M, Siow R, Moskalev A. Longevity medicine: upskilling the physicians of tomorrow. Lancet Healthy Longev. 2021; 2:e187–8. 10.1016/S2666-7568(21)00024-6 36098117

[162] Seals DR, Melov S. Translational geroscience: emphasizing function to achieve optimal longevity. Aging (Albany NY). 2014; 6:718–30. 10.18632/aging.100694 25324468 PMC4221919

[163] Newman JC, Sokoloski JL, Robbins PD, Niedernhofer LJ, Reed MJ, Wei J, Austad SN, Barzilai N, Cohen HJ, Kuchel GA, Kirkland JL, Pignolo RJ. Creating the Next Generation of Translational Geroscientists. J Am Geriatr Soc. 2019; 67:1934–9. 10.1111/jgs.16055 31287934 PMC6771814

[164] Moqri M, Herzog C, Poganik JR, Justice J, Belsky DW, Higgins-Chen A, Moskalev A, Fuellen G, Cohen AA, Bautmans I, Widschwendter M, Ding J, Fleming A, et al, and Biomarkers of Aging Consortium. Biomarkers of aging for the identification and evaluation of longevity interventions. Cell. 2023; 186:3758–75. 10.1016/j.cell.2023.08.003 37657418 PMC11088934

[165] Johnson AA, English BW, Shokhirev MN, Sinclair DA, Cuellar TL. Human age reversal: Fact or fiction? Aging Cell. 2022; 21:e13664. 10.1111/acel.13664 35778957 PMC9381899

[166] Heard DS, Tuttle CSL, Lautenschlager NT, Maier AB. Repurposing Proteostasis-Modifying Drugs to Prevent or Treat Age-Related Dementia: A Systematic Review. Front Physiol. 2018; 9:1520. 10.3389/fphys.2018.01520 30425653 PMC6218672

[167] Lee DJW, Hodzic Kuerec A, Maier AB. Targeting ageing with rapamycin and its derivatives in humans: a systematic review. Lancet Healthy Longev. 2024; 5:e152–62. 10.1016/S2666-7568(23)00258-1 38310895

[168] Levine ME, Lu AT, Quach A, Chen BH, Assimes TL, Bandinelli S, Hou L, Baccarelli AA, Stewart JD, Li Y, Whitsel EA, Wilson JG, Reiner AP, et al. An epigenetic biomarker of aging for lifespan and healthspan. Aging (Albany NY). 2018; 10:573–91. 10.18632/aging.101414 29676998 PMC5940111

[169] Ruckstuhl MM, Bischof E, Blatch D, Buhayer A, Goldhahn J, Battegay E, Tichelli A, Ewald CY. Translational longevity medicine: a Swiss perspective in an ageing country. Swiss Med Wkly. 2023; 153:40088. 10.57187/smw.2023.40088 37410895

[170] Bonnes SLR, Strauss T, Palmer AK, Hurt RT, Island L, Goshen A, Wang LYT, Kirkland JL, Bischof E, Maier AB. Establishing healthy longevity clinics in publicly funded hospitals. Geroscience. 2024; 46:4217–23. 10.1007/s11357-024-01132-0 38512582 PMC11336016

